# Opioid type, rather than liver function, is associated with postoperative nausea and vomiting after painless gastroscopy in decompensated cirrhosis

**DOI:** 10.3389/fphar.2026.1865501

**Published:** 2026-08-04

**Authors:** Xiao-Yu Gu, Jin-Feng Cao, Xin-Yang Zhang, Liu-Qin Jiang

**Affiliations:** Department of Anesthesiology, NO.215 Hospital of Shaanxi Nuclear Industry, Xianyang, China

**Keywords:** alfentanil, decompensated cirrhosis, nalbuphine, painless gastroscopy, postoperative nausea and vomiting

## Abstract

**Background:**

Postoperative nausea and vomiting (PONV) is common after painless gastroscopy. The independent predictive value of preoperative albumin, total bilirubin, Apfel score, and opioid type for PONV in decompensated cirrhosis is unknown.

**Objective:**

In this exploratory post-hoc secondary analysis of a double-blind randomized controlled trial, we aimed to determine whether preoperative albumin, total bilirubin, Apfel score, and opioid type (alfentanil vs. nalbuphine) independently predict PONV within 48 h after painless gastroscopy in decompensated cirrhosis, and to compare their effects on 24 h postoperative liver function.

**Results:**

Overall incidence of PONV was 24.00%, significantly higher with nalbuphine (P < 0.001). Univariate analysis: PONV patients were older (P = 0.012); no differences in preoperative albumin, total bilirubin, or Apfel score (all P > 0.05). Multivariable regression: preoperative albumin and total bilirubin were not independent predictors (both P > 0.05). Each 1-point increase in Apfel score gave (OR 1.40, 95% CI: 0.92–2.13; P = 0.116). Alfentanil significantly reduced PONV risk vs. nalbuphine (OR = 0.19, 95% CI: 0.08–0.47; P < 0.001); sensitivity analysis adjusted for age gave OR 0.20 (95% CI: 0.08–0.49; P < 0.001). Postoperatively, total bilirubin increased significantly in both groups (P < 0.05), whereas albumin showed no significant changes (P > 0.05). These mild fluctuations were comparable between the alfentanil and nalbuphine groups.

**Conclusion:**

Preoperative albumin, total bilirubin and Apfel score did not independently predict PONV. Alfentanil was independently associated with a significantly reduced risk of PONV compared to nalbuphine. Postoperative liver function changes were mild, similar between groups and unrelated to PONV. Our findings are hypothesis-generating and require confirmation in larger studies.

## 1 Background

Liver cirrhosis is a major global health concern, responsible for approximately 1 million deaths annually due to its complications (Luo et al., 2025; Wang et al., 2024). A key consequence of cirrhosis is portal hypertension, which promotes the development of esophageal and gastric varices. Because variceal hemorrhage is a life-threatening complication, regular gastroscopic surveillance is recommended in this population. Painless gastroscopy, which improves patient comfort and compliance, has become a routine clinical practice. In this study, “painless gastroscopy” refers to anesthesiologist-administered deep sedation with intravenous propofol and an opioid (detailed in Section 2.4 Study Protocol). However, impaired liver function can significantly alter pharmacokinetics, requiring extra caution in the selection of anesthetic agents (Bedewy and El-Kassas, 2023). Although routine liver function indicators are commonly considered when assessing perioperative risk, the inherent properties of anesthetic drugs may be equally important yet are sometimes underappreciated in this specific population.

In this population, postoperative nausea and vomiting (PONV) carries particular risks: retching and vomiting may precipitate rupture of esophagogastric varices, and altered drug metabolism may further modulate its occurrence. PONV is a common complication after anesthesia, with an incidence of approximately 20%–30%. It not only compromises the quality of postoperative recovery but may also lead to severe consequences such as aspiration and fluid-electrolyte disturbances (Gan et al., 2020). The classic PONV prediction tool, the Apfel score, incorporates four factors: female sex, non-smoking status, history of motion sickness/PONV, and postoperative opioid use (Apfel et al., 2002). The most recent 2025 consensus guidelines continue to endorse the simplified Apfel score as a practical risk stratification tool in the general surgical population (Gan et al., 2025). However, its validity in patients with cirrhosis has yet to be established.

Albumin and total bilirubin, as classic biomarkers of hepatic synthetic and excretory function, may theoretically modulate PONV risk by influencing drug protein binding and clearance (Duthaler et al., 2024; Nagar et al., 2024): patients with poorer hepatic functional reserve exhibit slower drug clearance, potentially increasing the risk of PONV. However, relevant clinical evidence remains lacking. Our previous randomized controlled trial demonstrated that alfentanil was associated with a significantly lower incidence of PONV compared with nalbuphine (Gu et al., 2025). Indeed, preoperative hypoalbuminemia was significantly associated with short-term adverse outcomes after partial hepatectomy, with the hypoalbuminemia group showing significantly increased postoperative complications and mortality (Kuhlmann et al., 2023). However, whether this difference is independent of patients’ hepatic functional reserve remains unclear. Therefore, this study conducted a post-hoc secondary analysis to investigate the independent predictive value of preoperative albumin, total bilirubin, Apfel score, and opioid type (alfentanil vs. nalbuphine) for PONV after painless gastroscopy in patients with decompensated cirrhosis, and to compare the effects of the two drugs on short-term postoperative liver function and its association with PONV.

## 2 Methods

### 2.1 Study design

This study is a post-hoc secondary analysis of a randomized controlled trial. During the original study, serum albumin and total bilirubin were prospectively collected but not analyzed in the primary report. Based on the observed between-group difference in PONV, we conducted this exploratory analysis to investigate the predictive value of these markers. The original trial was approved by the Institutional Review Board of the NO.215 Hospital of Shaanxi Nuclear Industry [Approval No. 2023 (012), Chairperson: Prof. Chaojie Ren, 18 September 2023] and registered with Chinese Clinical Trial Registry (registration number: ChiCTR2300077062). All participants provided written informed consent. This article follows the STROBE (Strengthening the Reporting of Observational Studies in Epidemiology) statement for the reporting of observational studies.

### 2.2 Study participants

**FIGURE 1:**
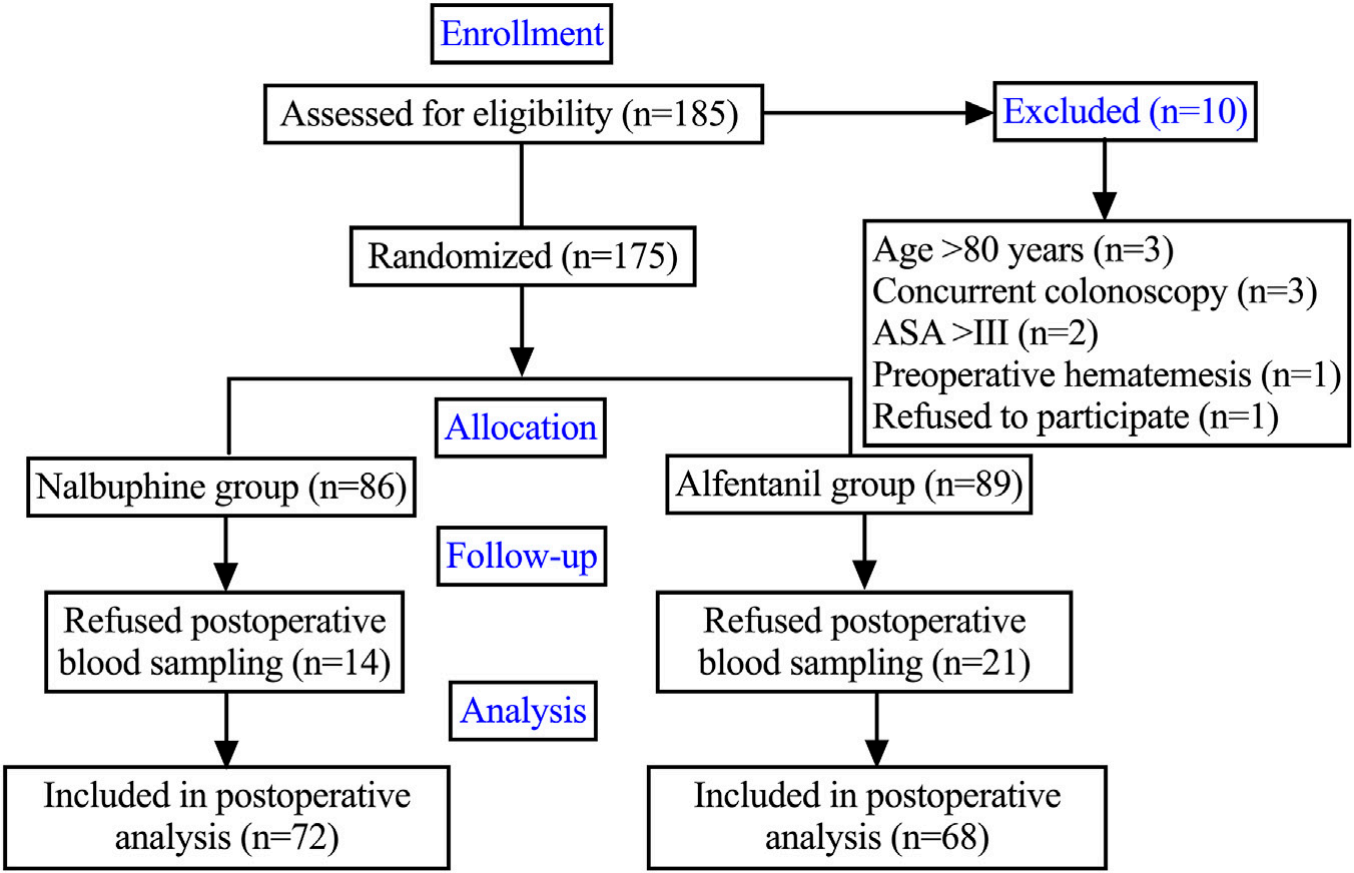
The flowchart of the study.

This original single-centre study included patients with decompensated cirrhosis scheduled for painless gastroscopy between November 2023 and December 2024 at NO.215 Hospital of Shaanxi Nuclear Industry, Xianyang, Shaanxi, China. A total of 185 patients were enrolled in the trial (Gu et al., 2025), of whom 175 completed the study (86 in the nalbuphine group and 89 in the alfentanil group). The patient flow diagram, including both the original trial enrollment and the selection process for the present secondary analysis, is presented in Figure 1.

### 2.3 Randomization and blinding

In the original RCT, patients were randomly assigned in a 1:1 ratio to the nalbuphine or alfentanil group using a computer-generated random number table. The allocation sequence was concealed in sealed envelopes and kept by nursing staff not involved in the study. All patients, anesthesiologists, and outcome assessors were blinded to group allocation. The analgesic drugs were prepared by nurses not involved in the study and were identified only by study number.

### 2.4 Study Protocol

The anesthetic protocol has been described in detail previously (Gu et al., 2025). Briefly, after routine monitoring and intravenous access, patients received either nalbuphine (0.1–0.15 mg/kg) or alfentanil (5–6 μg/kg), followed by titration of an etomidate-propofol mixture (1:1) to achieve a bispectral index (BIS) of 40–60 and loss of consciousness. Gastroscopy was then performed, with additional boluses given as needed to maintain adequate anesthetic depth. All patients were monitored in the post-anesthesia care unit (PACU) postoperatively. Adverse events within 48 h were recorded. All procedures were performed by the same endoscopist and anesthesiologist.

### 2.5 Outcomes

Based on the original RCT, the following additional data were collected for this study: preoperative and 24 h postoperative serum levels of albumin and total bilirubin, and the occurrence of PONV (nausea, vomiting, or both) within 48 h after the procedure. The Apfel simplified risk score was calculated by assigning 1 point for each of the following: female sex, non-smoking status, history of motion sickness or previous PONV, and postoperative opioid use. As all patients in this study received opioids, the possible score range was 1–4, with higher scores indicating greater predicted PONV risk. The primary outcome was the incidence of PONV. Secondary outcomes included postoperative changes in liver function (Δalbumin and Δtotal bilirubin) and their association with PONV.

### 2.6 Statistical analysis

Statistical analyses were performed using SPSS version 27.0. Continuous variables were tested for normality using the Shapiro–Wilk test. Normally distributed data are presented as mean ± standard deviation and were compared between groups using the independent samples t-test. Non-normally distributed data are presented as median (interquartile range, IQR) and were compared using the Mann–Whitney U test. Categorical variables were analyzed using the Chi-square test or Fisher’s exact test, as appropriate.

For the multivariable logistic regression analysis, based on clinical relevance and theoretical considerations, the primary model included the following independent variables: opioid type (alfentanil vs. nalbuphine), Apfel score, preoperative albumin, and preoperative total bilirubin. Results are presented as odds ratios (OR) with 95% confidence intervals (CI). Model calibration was assessed using the Hosmer–Lemeshow goodness-of-fit test, where a non-significant result (P > 0.05) indicates acceptable calibration. Multicollinearity among independent variables was assessed by calculating variance inflation factors (VIF) prior to regression analysis; all VIF values were less than 5, indicating no serious multicollinearity.

For sensitivity analysis, an additional regression model including age was constructed to evaluate its impact on the main findings. Although age was significantly associated with PONV in univariate analysis, it was not included in the primary model to maintain model stability, given the number of PONV events (n = 42) and the rule of thumb of at least 10 events per variable.

### 2.7 Handling of missing data

Of the 175 enrolled patients, preoperative variables (including albumin and total bilirubin) were complete, whereas the postoperative liver function data were missing for 35 patients due to refusal to undergo blood sampling (27/35) or laboratory errors (8/35). To evaluate the impact of missing data, baseline characteristics were compared between patients with (n = 140) and without (n = 35) postoperative data (Supplementary Table). No significant differences were observed between the two groups in age, BMI, or preoperative total bilirubin (all P > 0.05). However, significant differences were found in PONV incidence (27.9% in the group with postoperative data vs. 8.6% in the group without, P = 0.017), preoperative albumin (P = 0.035), Apfel score (P = 0.046), and history of motion sickness (P = 0.022). These findings indicate that the missingness was not completely at random but was associated with both the outcome and several predictors.

To minimize potential selection bias and preserve statistical power, multiple imputation (MI) was performed as the primary analytical approach. Missing values were imputed using the fully conditional specification method with 10 iterations. The imputation model included: age, sex, BMI, smoking status, history of motion sickness, opioid type, Apfel score, PONV occurrence, preoperative albumin, preoperative total bilirubin, postoperative albumin, and postoperative total bilirubin. According to current methodological guidelines recommending that the number of imputations be at least equal to the percentage of incomplete cases (White et al., 2011), we generated 20 multiply imputed datasets (approximately 20% missingness). Rubin’s rules were applied to combine parameter estimates (mean differences) and their standard errors to obtain valid statistical inferences (Austin et al., 2025). All subsequent inferential analyses for postoperative liver function were conducted on the MI-pooled dataset (effective n = 175). Specifically, paired-samples t-tests were used for within-group comparisons of preoperative and postoperative values, and independent-samples t-tests were used for between-group comparisons of changes (Δ) between the two groups. For descriptive purposes, preoperative and postoperative values are presented as median (interquartile range) based on the original complete cases (n = 140) to reflect the true clinical distribution. All reported mean differences, 95% confidence intervals (CIs), and P-values are derived from the pooled MI analyses. A two-sided P value < 0.05 was considered statistically significant.

## 3 Patient characteristics

A total of 175 patients were included in the analysis, with 86 in the nalbuphine group and 89 in the alfentanil group. The overall incidence of PONV was 24.0% (42/175), with 32 cases (37.2%) in the nalbuphine group and 10 cases (11.2%) in the alfentanil group (P < 0.001). Table 1 presents the baseline characteristics of patients stratified by PONV occurrence. Patients in the PONV group were significantly older than those in the non-PONV group (62.0 ± 10.1 years vs. 57.1 ± 11.4 years, P = 0.012). No significant differences were observed between the two groups in sex, BMI, procedure type, smoking status, history of motion sickness, preoperative albumin, preoperative total bilirubin, or Apfel score (all P > 0.05). Notably, the proportion of patients receiving nalbuphine was significantly higher in the PONV group than in the non-PONV group (76.2% vs. 23.8%, P < 0.001).

**TABLE 1:** Baseline characteristics of patients with complete vs. missing postoperative liver function data.

Variable	Complete data (n = 140)	Missing data (n = 35)	P value
Preoperative albumin (g/L), median (IQR)	37.00 (32.50–40.80)	36.80 (32.10–40.20)	0.035^*^
PONV incidence, n (%)	39.00 (27.86)	3.00 (8.57)	0.017^*^
Apfel score, median (IQR)	2.00 (1.00–3.00)	2.00 (1.00–3.00)	0.046^*^
History of motion sickness, n (%)	10.00 (7.14)	7.00 (20.00)	0.022^*^

No significant differences were observed between the two groups in age, sex, preoperative total bilirubin, or opioid group allocation (all P > 0.05). Values are median (IQR) for continuous variables and n (%) for categorical variables. * means P < 0.05.

Abbreviations: PONV, postoperative nausea and vomiting.

Baseline characteristics of patients stratified by opioid group are shown in Table 2. Preoperative total bilirubin was significantly higher in the alfentanil group than in the nalbuphine group [median (IQR): 21.95 (14.03–36.10) μmol/L vs. 17.20 (12.23–25.78) μmol/L, P = 0.020], while preoperative albumin did not differ significantly between the two groups (P = 0.336). This difference was adjusted for as a covariate in the multivariable regression analysis.

**TABLE 2:** Preoperative baseline characteristics by analgesic group (n = 175).

Variable	Alfentanil group (n = 89)	Nalbuphine group (n = 86)	*P* value
Age (years), *x̄* ± *s*	58.46 ± 11.16	57.87 ± 11.53	0.721
Female, n (%)	44 (49.44)	43 (50.00)	0.876
BMI (kg/m^2^), median (IQR)	23.60 (21.90–25.80)	23.70 (22.00–25.90)	0.813
Apfel score, median (IQR)	2.00 (1.00–3.00)	2.00 (1.00–3.00)	0.846
Preoperative albumin (g/L), median (IQR)	36.75 (32.40–39.68)	37.00 (31.73–42.18)	0.336
Preoperative total bilirubin (μmol/L), median (IQR)	21.95 (14.03–36.10)	17.20 (12.23–25.78)	0.020^*^

Continuous variables are presented as mean ± standard deviation (SD) or median with interquartile range (IQR). Categorical variables are presented as number (percentage). P values were calculated using independent t-test, Mann-Whitney U test, or Chi-square test as appropriate. * means P < 0.05.

Abbreviations: BMI, body mass index.

## 4 Results

### 4.1 Multivariable logistic regression analysis for PONV

**FIGURE 2:**
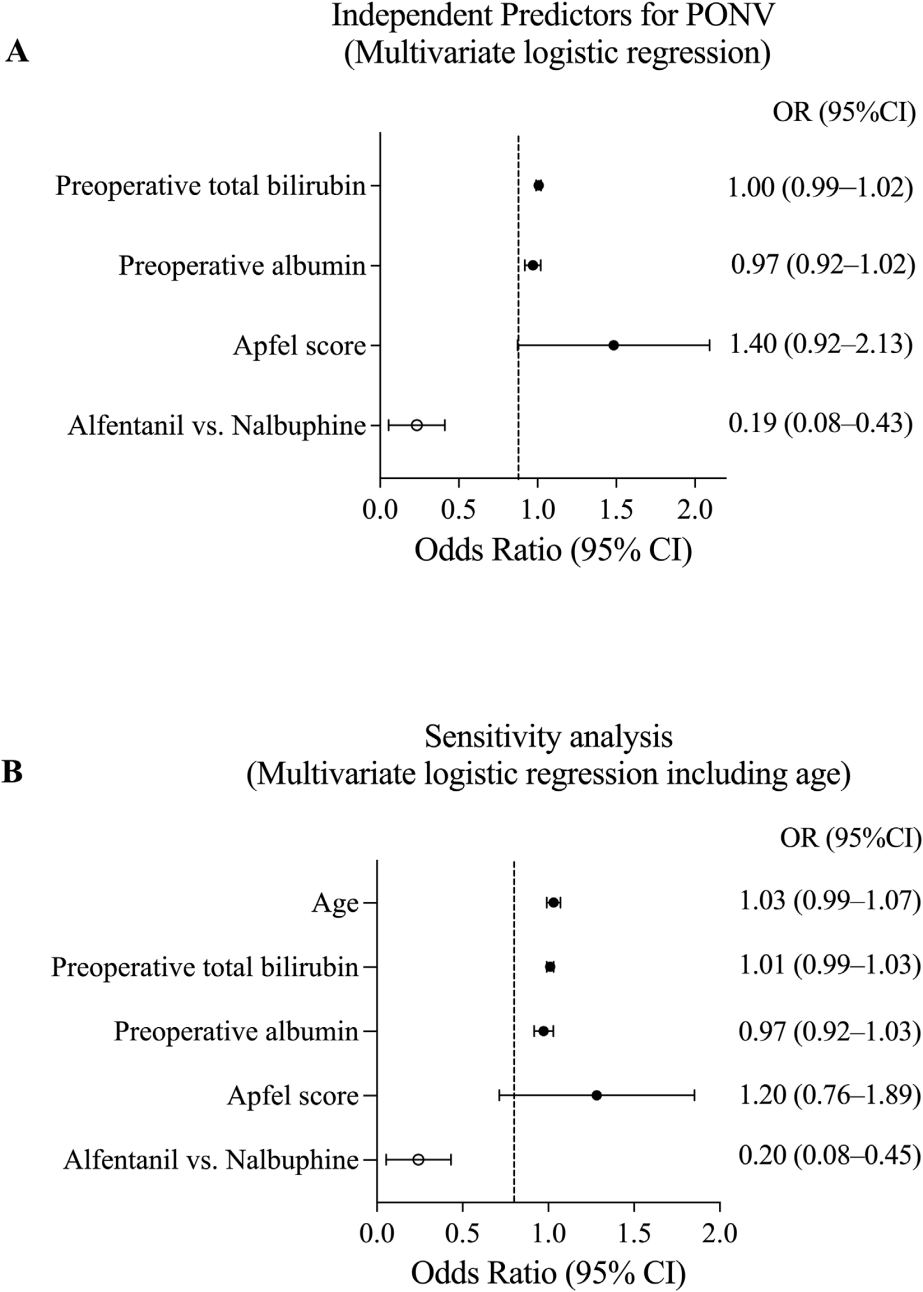
Forest plots of multivariate logistic regression analyses for independent predictors of PONV. **(A)** Primary regression model without age adjustment. Solid black circles indicate odds ratio (OR) point estimates for non-significant predictors (95% confidence intervals [CIs] cross OR = 1.0); open circles represent statistically significant predictors whose 95% CIs do not overlap the null line at OR = 1.0. Horizontal bars show 95% CIs, and OR together with 95% CI values are listed to the right of each variable as OR (lower bound–upper bound). **(B)** Sensitivity analysis with age added as a covariate to verify the stability of predictive factors identified in the primary model, with identical graphical notation as panel **(A)**. Abbreviations: CI, confidence interval; OR, odds ratio; PONV, postoperative nausea and vomiting.

The results of the primary multivariable logistic regression analysis are presented in Table 3 and Figure 2A. After adjusting for other factors, preoperative albumin (OR = 0.97, 95% CI: 0.92–1.02, P = 0.211) and total bilirubin (OR = 1.01, 95% CI: 0.99–1.02, P = 0.315) were not independently associated with PONV. Each one-point increase in the Apfel score was associated with a 40.1% increase in the risk of PONV, although this did not reach statistical significance (OR = 1.40, 95% CI: 0.92–2.13, P = 0.116). In contrast, alfentanil, compared with nalbuphine, significantly reduced the risk of PONV (OR = 0.19, 95% CI: 0.08–0.43, P < 0.001). The Hosmer-Lemeshow goodness-of-fit test indicated acceptable calibration of the model (χ^2^ = 3.82, P = 0.873).

**TABLE 3:** Primary multivariate logistic regression analysis for PONV (n = 175).

Variable	β	SE	Wald χ^2^	OR (95%CI)	P value
Alfentanil (vs. nalbuphine)	−1.67	0.42	15.58	0.19 (0.08–0.43)	<0.001^**^
Apfel score (per 1-point increase)	0.34	0.21	2.48	1.40 (0.92–2.13)	0.116
Preoperative albumin (g/L)	−0.03	0.03	1.57	0.97 (0.92–1.02)	0.211
Preoperative total bilirubin (μmol/L)	0.01	0.01	1.01	1.00 (0.99–1.02)	0.315

Multicollinearity diagnostics showed VIF, values ranging from 1.02 to 1.06, indicating no significant multicollinearity. ** means P < 0.01.

Abbreviations: OR, odds ratio; CI, confidence interval; β, regression coefficient; SE, standard error.

### 4.2 Sensitivity analysis

When age was added to the multivariable regression model (Table 4, Figure 2B), the protective effect of alfentanil remained significant (OR = 0.20, 95% CI: 0.08–0.45, P < 0.001), consistent with the findings of the primary analysis. The effect of the Apfel score was attenuated (OR = 1.20, 95% CI: 0.76–1.89, P = 0.438), potentially because age shares variance with certain components of the score, thereby partially accounting for the risk originally attributed to it. Age itself was not independently associated with PONV (OR = 1.03, 95% CI: 0.99–1.07, P = 0.089). These results demonstrate the robustness of our primary conclusion that alfentanil is a strong protective factor against PONV.

**TABLE 4:** Sensitivity analysis: Multivariable logistic regression including age (n = 175).

Variable	β	SE	Wald χ^2^	OR (95%CI)	P value
Alfentanil (vs. nalbuphine)	−1.64	0.43	14.68	0.20 (0.08–0.45)	<0.001**
Apfel score (per 1-point increase)	0.18	0.23	0.60	1.20 (0.76–1.89)	0.438
Age (per 1-year increase)	0.03	0.02	2.90	1.03 (0.99–1.07)	0.089
Preoperative albumin (g/L)	−0.03	0.03	1.14	0.97 (0.92–1.03)	0.286
Preoperative total bilirubin (μmol/L)	0.01	0.01	1.27	1.01 (0.99–1.03)	0.261

This sensitivity analysis added age as a covariate to the primary model (Table 3). Multicollinearity diagnostics showed all variance inflation factor (VIF) values <1.1. ** means P < 0.01.

Abbreviations: OR, odds ratio; CI, confidence interval; β, regression coefficient; SE, standard error.

### 4.3 Postoperative changes in liver function

Based on the pooled analysis of the multiply imputed datasets (n = 175) (Table 5), albumin levels did not change significantly postoperatively in either the alfentanil (P = 0.743) or the nalbuphine group (P = 0.141), and the difference in Δalbumin between groups was not statistically significant (P = 0.285). Conversely, both the alfentanil (P = 0.013) and nalbuphine (P = 0.001) groups demonstrated a significant postoperative increase in total bilirubin, with no significant difference in the magnitude of change (ΔTBIL) between the two groups (P = 0.877).

**TABLE 5:** Perioperative changes in liver function markers using multiple imputation datasets.

Parameter	Group	Preoperative (n = 175)	Postoperative (n = 140)	^a^ΔMean difference (95% CI)	^a^Intra-group P value	^b^Mean difference between groups (95% CI)	^b^Inter-group P value
Albumin (g/L)	Alfentanil group	34.70 (33.35–40.00)	35.65 (31.63–39.30)	−0.22 (−1.52–1.09)	0.743	1.24 (−1.03–3.50)	0.285
​	Nalbuphine group	37.70 (31.88–42.53)	35.95 (32.23–40.53)	−1.45 (−3.39–0.49)	0.142	​	​
Total bilirubin (μmol/L)	Alfentanil group	19.30 (14.70–35.15)	26.00 (18.78–43.13)	4.10 (0.90–7.31)	0.013^*^	−0.31 (−4.28–3.65)	0.877
​	Nalbuphine group	16.85 (11.97–24.88)	23.65 (16.33–29.38)	4.42 (1.83–7.01)	0.001^**^	​	​

Preoperative and postoperative values are presented as median (interquartile range) based on original complete cases (n = 140) to reflect true clinical distribution. Mean differences, 95% Confidence Intervals (CI), and P-values are derived from pooled analyses of 20 multiply imputed datasets (n = 175).

^a^ Within-group changes were assessed by paired *t*-test, with mean difference calculated as postoperative value minus preoperative value.

^b^ Between-group differences in the magnitude of change (alfentanil vs. nalbuphine) were compared using independent *t*-test. * means P < 0.05, ** means P < 0.01.

## 5 Discussion

This study conducted a post-hoc secondary analysis of a randomized controlled trial, with the following main findings: First, preoperative albumin and total bilirubin did not independently predict PONV after painless gastroscopy in patients with decompensated cirrhosis. Second, the Apfel score did not demonstrate significant predictive value in this study population. Third, alfentanil significantly reduced the risk of PONV compared with nalbuphine, an advantage that remained highly significant after adjusting for between-group differences in preoperative total bilirubin and age, indicating that alfentanil shows a strong independent protective association with PONV in this population. Fourth, both anesthetic regimens had mild and directionally consistent effects on 24-h postoperative liver function, with no clear association with PONV.

Albumin and total bilirubin are classic biomarkers of hepatic synthetic and excretory function. Theoretically, patients with diminished hepatic functional reserve may exhibit impaired drug clearance, potentially elevating the risk of PONV. However, the present study identified no independent association between these preoperative biochemical markers and PONV. Several factors may underpin this discrepancy. First, the liver possesses substantial functional redundancy; clinically significant impairment typically manifests only after extensive parenchymal injury. In our cohort, most patients maintained relatively compensated hepatic function despite their diagnosis, and the resulting narrow range of these markers may have lacked the granularity required to discriminate PONV risk. Second, preoperative biochemical tests primarily reflect hepatic status at a single time point and do not necessarily quantify the dynamic activity of specific metabolic pathways relevant to opioid clearance (Balyan et al., 2017; Gan et al., 2025; Apfel et al., 1999). Third, our limited sample size (42 PONV events) may have constrained the statistical power to detect subtle associations, potentially leading to a type II error. Despite these findings, this result holds clinical significance: it suggests that routine liver function tests may have limited independent predictive value in PONV risk stratification for cirrhotic patients. In this population, greater attention may be warranted toward established clinical risk scores and the judicious selection of intraoperative opioids.

This study also observed that the Apfel simplified risk score did not reach statistical significance in either univariate (P = 0.143) or multivariable (P = 0.116) analyses, contrasting with findings in general population studies. Although not statistically significant, the point estimate was compatible with an increased risk, but the wide confidence interval crossing 1 precludes firm conclusions. Several factors may explain the suboptimal predictive performance of the Apfel score in this cohort. First, the inherent discriminative ability of the score was likely reduced by the clinical setting. As all patients in this study received postoperative opioids, this factor contributed 1 point to every participant, effectively narrowing the score range to 1-4 and diminishing the model’s sensitivity. This finding is consistent with a recent study in patients with liver cancer, where the Apfel score showed poor discrimination and calibration, highlighting the need for disease-specific PONV risk stratification tools (Chen et al., 2023). Second, a “masking effect” of pharmacological intervention may have occurred. Our multivariable model demonstrated that the choice of opioid (alfentanil vs. nalbuphine) was a far more potent predictor of outcome than baseline characteristics. This dominant pharmacological influence may have overshadowed the predictive value of sex and smoking status, which are central to the Apfel score (Gan et al., 2020). Third, and importantly, as a post-hoc secondary analysis, this study was not specifically powered to validate the Apfel score. The limited number of PONV events (n = 42) may have led to insufficient statistical power to detect a modest effect, and the absence of statistical significance should therefore not be definitively interpreted as evidence of poor performance.

Consequently, while the classic Apfel score should be applied with appropriate caution in patients with decompensated cirrhosis, our findings suggest that, in this specific population, opioid selection may represent a more critical and actionable risk management strategy than relying solely on conventional risk scores. Future studies with adequate power and prospective validation are needed to clarify the true predictive value of the Apfel score in cirrhotic patients.

In contrast to the limited predictive value of preoperative liver function markers and the Apfel score, the choice of opioid emerged as a powerful determinant of PONV risk. Our findings demonstrate a robust advantage of alfentanil over nalbuphine, an effect that remained highly significant after adjusting for potential confounders. This pronounced clinical disparity is likely rooted in the distinct receptor pharmacology and pharmacokinetic profiles of the two agents. Nalbuphine, as a κ-receptor agonist, may directly stimulate the chemoreceptor trigger zone and induce dysphoria, which can exacerbate the subjective perception of nausea (Smith and Laufer, 2014; Holden et al., 2005; Gao et al., 2022). In contrast, alfentanil is a pure μ-receptor agonist and avoids this κ-mediated emetic component. In addition, alfentanil is characterized by exceptionally rapid redistribution and clearance, which may limit sustained chemoreceptor trigger zone activation during short procedures, even in patients with altered hepatic metabolism (Camu et al., 1982; El-Khateeb et al., 2020). Accordingly, the robust protective effect of alfentanil (OR<0.2) underscores that opioid selection contributes substantially to PONV prevention in this population.

In the present study, overall, postoperative total bilirubin levels increased significantly, while albumin levels showed no statistically significant change. The observed mild elevation in total bilirubin, while statistically significant, remained within a reasonable physiological range and did not meet criteria for acute liver injury. This might be partly explained by a nonspecific procedural stress response or by transient, subclinical alterations in biliary excretion. Although postoperative liver function fluctuations are well-documented following major abdominal or hepatic surgery (Mladinov et al., 2022; Chen et al., 2025), it is unclear whether these mechanisms are generalizable to minimally invasive procedures like gastroscopy. Given its small magnitude, the clinical relevance of this change is likely negligible. Postoperative albumin levels did not change significantly in either group after multiple imputation. Although the complete-case (n = 140) analysis had suggested a decrease in the nalbuphine group, this discrepancy is attributable to the non-random missingness: patients missing follow-up data had lower preoperative albumin and lower PONV incidence, and their exclusion biased the complete-case estimate. Multiple imputation corrected this bias, yielding a more reliable conclusion that neither opioid adversely affects short-term albumin levels. This finding is reassuring, as concerns regarding opioid-induced hepatic dysfunction in patients with cirrhosis remain a topic of ongoing investigation (Mladinov et al., 2022; Rubin et al., 2025).

Taken together, these findings provide evidence for the short-term hepatic safety of both anesthetic regimens in patients with cirrhosis. The mild and transient nature of the observed changes, coupled with their lack of association with PONV, supports the clinical utility of both alfentanil and nalbuphine for painless gastroscopy in this vulnerable population.

Synthesizing the findings of this study, the following recommendations can be made for the management of PONV in patients with decompensated cirrhosis undergoing painless gastroscopy: (1) when assessing preoperative PONV risk, routine liver function tests such as albumin and total bilirubin need not be overemphasized; (2) regarding opioid selection, alfentanil is associated with a significantly lower risk of PONV compared with nalbuphine and may represent a preferable analgesic option in this population; and (3) both drugs have minimal and comparable effects on liver function, supporting their safe use in clinical practice. Our finding that routine liver function tests lack independent predictive value aligns with the recently published ACG guideline on perioperative management of cirrhosis, which emphasizes the need for cirrhosis-specific risk calculators rather than relying solely on standard biochemical markers (Mahmud et al., 2025).

This study has several limitations. First, it was a single-center study involving short-duration procedures performed by the same endoscopist and anesthesiologist, which may limit generalizability. For instance, longer procedures or those requiring repeated opioid dosing could attenuate the PONV-suppressive advantage of alfentanil, which partly relies on its rapid redistribution. Different anesthetic protocols (e.g., propofol without an opioid, or co-administration with remifentanil or fentanyl) may also alter the relative risk profiles. Patients with more advanced hepatic dysfunction could exhibit substantially impaired drug clearance, potentially modifying both efficacy and safety. Non-endoscopic procedures such as abdominal surgery involve greater physiological trespass, and the minimal hepatic impact observed here may not directly translate to those settings. Whether our findings extend to these scenarios remains to be determined. Second, as a post-hoc secondary analysis, the sample size was based on the original trial and may have been insufficient to detect modest effects of liver function markers and the Apfel score. Third, postoperative follow-up was limited to 24 h, precluding assessment of longer-term changes in liver function. Fourth, postoperative liver function data were missing in 20% of patients (35/175). Although we rigorously employed multiple imputation to mitigate potential selection bias, this statistical technique inherently relies on the assumption that data are missing at random (MAR) conditionally on the variables included in the model, and residual bias from unmeasured confounders cannot be entirely ruled out. Fifth, there was a chance imbalance in preoperative total bilirubin between the two groups (P = 0.020), which, although adjusted for in the multivariable models, may still indicate incomplete randomization. Sixth, with 42 PONV events, our multivariable model may be subject to limited precision, as reflected by the relatively wide confidence intervals for certain predictors (e.g., Apfel score: OR 1.401, 95% CI 0.92–2.13). Although we adhered to the rule of thumb of maintaining at least 10 events per variable (EPV ≈10.5) to minimize this risk, the estimates remain somewhat unstable. Given these statistical limitations and the post-hoc exploratory design, our findings are hypothesis-generating only; thus, future multicenter, large-sample studies are warranted to validate our findings and provide more robust estimates. Further research exploring the pharmacogenomic mechanisms underlying PONV, as well as interventional studies evaluating the efficacy of prophylactic antiemetics based on risk stratification, is also needed.

## 6 Conclusion

Preoperative albumin and total bilirubin were not independent predictors of PONV after painless gastroscopy in patients with decompensated cirrhosis. The Apfel score did not reach statistical significance in this study population but showed a clinically meaningful trend. Alfentanil significantly reduced the risk of PONV compared with nalbuphine, showing a strong independent protective association with PONV in this population, with robust results. Both sedation regimens had similar and mild effects on short-term postoperative liver function. In clinical practice, alfentanil may be preferred as an analgesic to reduce the risk of PONV in this patient population. These findings should be considered hypothesis-generating rather than confirmatory. External validation in larger, prospective multi-center cohorts is needed before drawing definitive conclusions.

## Data Availability

The datasets presented in this article are not readily available due to patient confidentiality and institutional privacy policies. Requests to access the datasets should be directed to the corresponding author.
