# Supplementary material for: Opioid type, rather than liver function, is associated with postoperative nausea and vomiting after painless gastroscopy in decompensated cirrhosis
Source: Front Pharmacol. 2026 Aug 4;17:1865501. doi: 10.3389/fphar.2026.1865501 (PMC13481240; doi:10.3389/fphar.2026.1865501)
Supplement: Supplementary file 1 [file Table1.docx]

Supplementary Table. Baseline characteristics of patients with complete vs. missing postoperative liver function data

| Variable | Complete data (n=140) | Missing data (n=35) | P value |
| --- | --- | --- | --- |
| Preoperative albumin (g/L), Median (IQR) | 37.00 (32.50–40.80) | 36.80 (32.10–40.20) | 0.035^*^ |
| PONV incidence, n (%) | 39.00 (27.86) | 3.00 (8.57) | 0.017^*^ |
| Apfel score, Median (IQR) | 2.00 (1.00–3.00) | 2.00 (1.00–3.00) | 0.046^*^ |
| History of motion sickness, n (%) | 10.00 (7.14) | 7.00 (20.00) | 0.022^*^ |

No significant differences were observed between the two groups in age, sex, preoperative total bilirubin, or opioid group allocation (all P > 0.05). Values are median (IQR) for continuous variables and n (%) for categorical variables. * means P<0.05.

Abbreviations: PONV, postoperative nausea and vomiting.
